# Correction: Spatial variation and antecedent sea surface temperature conditions influence Hawaiian intertidal community structure

**DOI:** 10.1371/journal.pone.0295828

**Published:** 2023-12-08

**Authors:** Rebecca J. Ward, T. Erin Cox, Steven L. Colbert, Anuschka Faucci, Florybeth Flores La Valle, Joanna Philippoff, Jessica L. B. Schaefer, Tracy N. Wiegner, Ian M. Ware, Matthew L. Knope

Steven L. Colbert should be listed as the third author, and his affiliation is 9: Marine Science Department, University of Hawaiʻi at Hilo, 200 W. Kawili St., Hilo, HI 96720. The contributions of this author are as follows: Conceptualization, Data Curation, Formal Analysis, Methodology, Supervision, Validation and Writing–Review & Editing.

Tracy N. Wiegner should be listed as the eighth author and affiliated with 9: Marine Science Department, University of Hawaiʻi at Hilo, 200 W. Kawili St., Hilo, HI 96720. The contributions of this author are as follows: Conceptualization, Data Curation, Formal Analysis, Methodology, Supervision, Validation and Writing–Review & Editing.

The correct citation is: Ward RJ, Cox TE, Colbert SL, Faucci A, La Valle FF, Philippoff J, et al. (2023) Spatial variation and antecedent sea surface temperature conditions influence Hawaiian intertidal community structure. PLoS ONE 18(6): e0286136. https://doi.org/10.1371/journal.pone.0286136
