# Supplementary figures and images for: Spatial variation and antecedent sea surface temperature conditions influence Hawaiian intertidal community structure
Source: PLoS One. 2023 Jun 2;18(6):e0286136. doi: 10.1371/journal.pone.0286136 (PMC10237483; doi:10.1371/journal.pone.0286136)

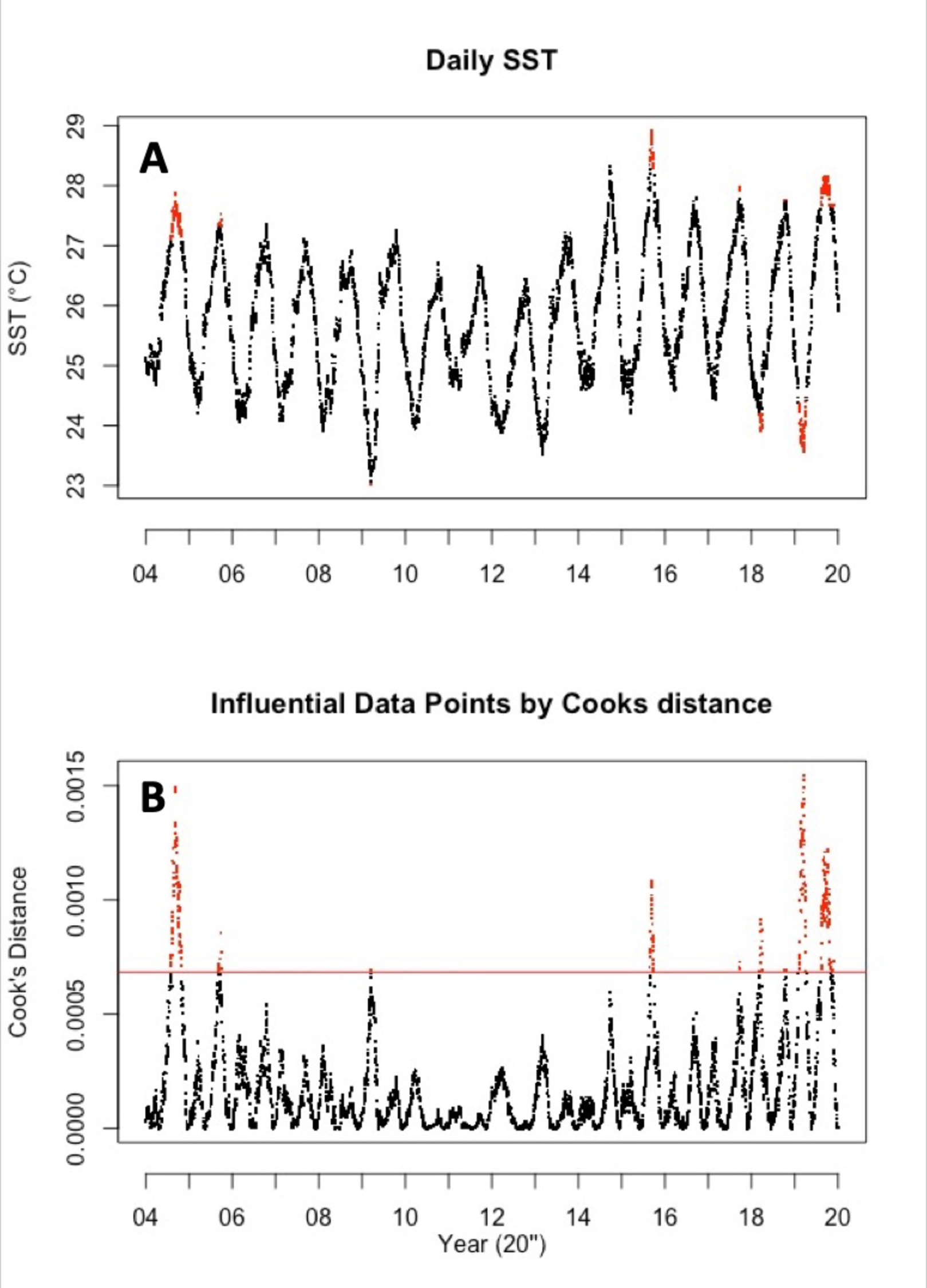

Supplement: S1 Fig — Influential data points (indicated as red dots) were identified via Cook’s Distance, and all observations with a Cook’s D value greater than 4/n (indicated as the red line, where n is the number of observations) are highlighted in red. For comparative purposes, Cook’s D plot (B) is stacked beneath a plot of daily SST values (A). Influential data points were identified as the highest summer SST highs in 2004, 2005, 2015, 2017, and 2019 and the lowest winter SST lows in 2009, 2018, and 2019. (TIF) [file pone.0286136.s001.tif]

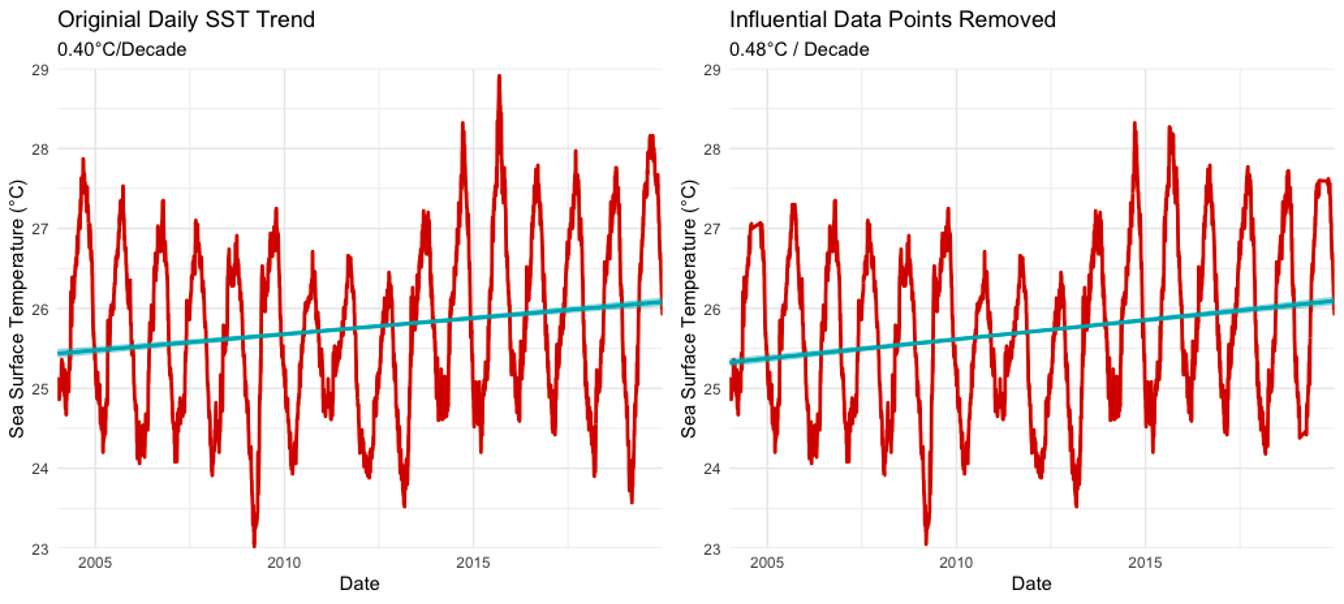

Supplement: S2 Fig — The original rate of warming with daily SST on the left, and the plot on the right has influential data points removed. Without the influential points (0.48°C Decade-1), the rate of warming is slightly greater than with them included (0.40°C Decade-1). (TIF) [file pone.0286136.s002.tif]

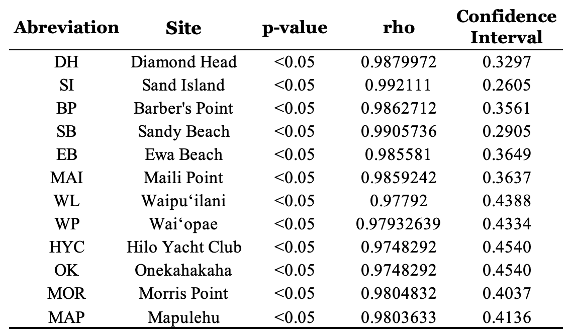

Supplement: S2 Table — (PNG) [file pone.0286136.s004.png]

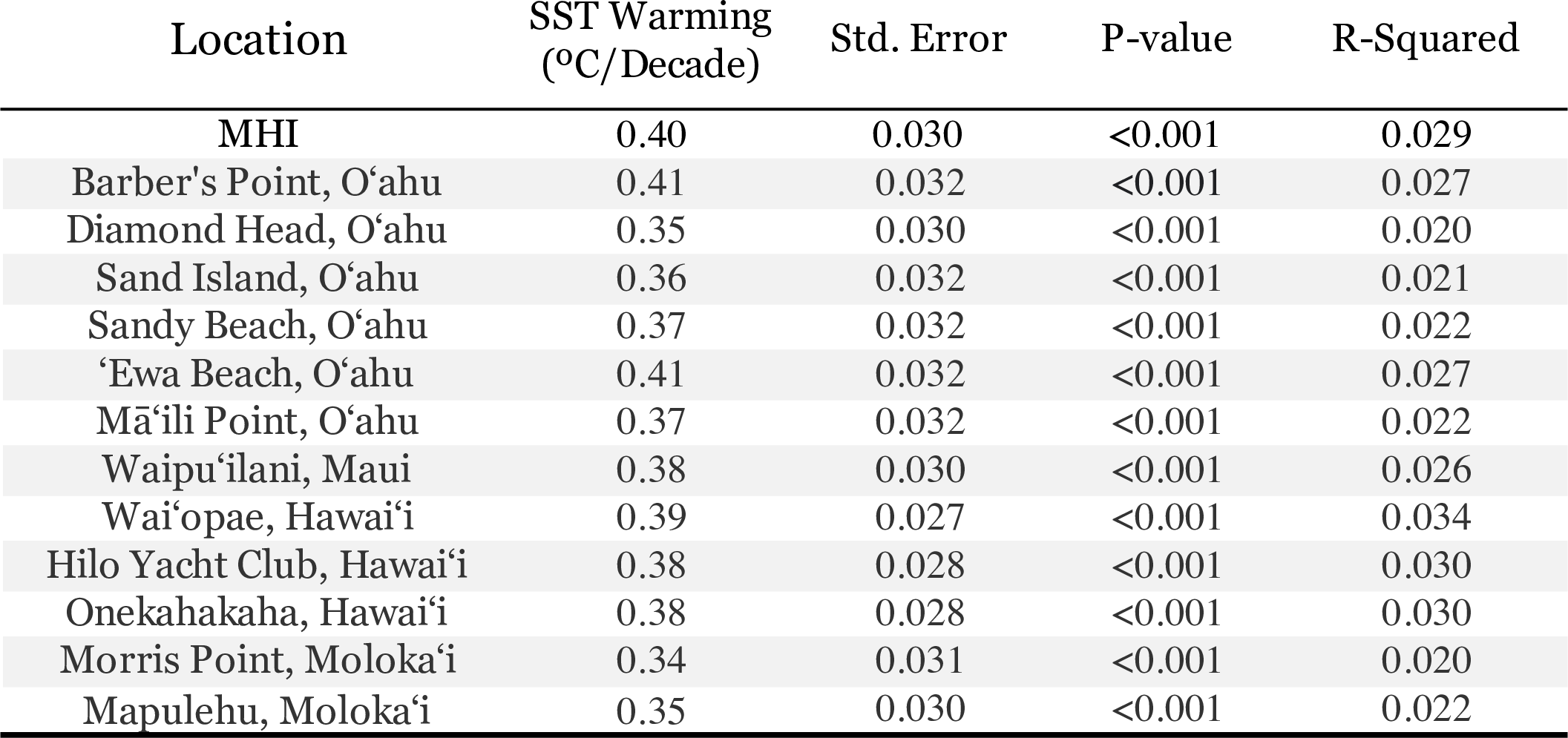

Supplement: S4 Table — (TIF) [file pone.0286136.s006.tif]
