# Supplementary material for: Spatial variation and antecedent sea surface temperature conditions influence Hawaiian intertidal community structure
Source: PLoS One. 2023 Jun 2;18(6):e0286136. doi: 10.1371/journal.pone.0286136 (PMC10237483; doi:10.1371/journal.pone.0286136)
Supplement: S1 Table — (A-E) Classifications of all algae and invertebrates documented in the Our Project in Hawai‘i’s Intertidal (OPIHI) database. Highly cryptic or extremely rare species were grouped at higher taxonomic levels to be conservative with respect to identification, while easily identifiable or highly abundant species were retained at the species level. Additionally, taxa were separated into organism type, group, and invasive classification (i.e., native, native-dominant, non-native invasive, and unknown). ‘Native-dominant’ categorizes algae or invertebrates which are native to the Hawaiian Islands but considered highly noxious and have been recognized for their potential to dominate Hawaiian habitats. The ‘non-native invasive’ classification refers to organisms often with high tolerances to disturbance and thermal variation that introduced to the Hawaiian Islands and are currently listed as invasive species by Hawaiʻi’s Division of Aquatic Resources (DAR 2020). The classification, ‘native’ is for organisms introduced the Hawaiian Islands which are not known to be noxious or dominant in Hawaiian intertidal habitats. Species with ambiguous identifications were classified as ‘unknown’. (PDF) [file pone.0286136.s003.pdf]

A

Initial Identification	Reclassified by Taxa	Classification
Acanthophora.spicifera	Acanthophora.spicifera	non-native.invasive
Ahnfeltiopsis.concinna	Ahnfeltiopsis.concinna	native
Ahnfeltiopsis.flabelliformis	Ahnfeltiopsis.flabelliformis	native
Amansia.glomerata	Amansia.glomerata	native
Asparagopsis.taxiformis	Asparagopsis.taxiformis	native
Asteronema.breviarticulatum	Asteronema.breviarticulatum	native
Avrainvillea.amadelpha	Avrainvillea.amadelpha	non-native.invasive
Boodlea.composita	Boodlea.composita	native
Bornetella.sphaerica	Bornetella.spp	native
Bornetella.spp	Bornetella.spp	native
Botryocladia.spp	Botryocladia.spp	native
Brown.Crust	Brown.Crust	unknown
Bryopsis.spp	Bryopsis.spp	native.dominant
Caulerpa.racemosa	Caulerpa.spp	native
Caulerpa.sertularioides	Caulerpa.spp	native
Caulerpa.spp	Caulerpa.spp	native
Centroceras.clavulatum	Centroceras.clavulatum	native
Chaetomorpha.antennina	Chaetomorpha.spp	native
Chaetomorpha.spp	Chaetomorpha.spp	native
Champia.parvula	Champia.parvula	native
Chnoospora.minima	Chnoospora.spp	native
Chnoospora.spp	Chnoospora.spp	native
Chondria.spp	Chondria.spp	non-native.invasive
Cladophora.spp	Cladophora.spp	native.dominant
Cladophora/Cladophoropsis	Cladophora.spp	native.dominant
Codium.arabicum	Codium.spp	native
Codium.edule	Codium.spp	native
Codium.spp	Codium.spp	native
Coelothrix.irregularis	Coelothrix.spp	native
Coelothrix.spp	Coelothrix.spp	native
Colpomenia.sinuosa	Colpomenia.sinuosa	native
Crounia	Crouania.spp	native
Crustose.coralline.algae	Crustose.coralline.algae	native
Blue-green.algae.(Lyngbya)	Cyanobacteria.spp	unknown
Cyanobacteria.spp	Cyanobacteria.spp	unknown
Lyngbya.majuscula	Cyanobacteria.spp	unknown
Unknown.cyanobacteria	Cyanobacteria.spp	unknown
Dictyopteris.spp	Dictyopteris.spp	native
Dictyosphaeria.cavernosa	Dictyosphaeria.spp	native.dominant
Dictyosphaeria.spp	Dictyosphaeria.spp	native.dominant
Dictyosphaeria.versluysii	Dictyosphaeria.spp	native.dominant
Dictyota.acutiloba	Dictyota.spp	native

B

Initial Identification	Reclassified by Taxa	Classification
Dictyota.sandvicensis	Dictyota.spp	native
Dictyota.spp	Dictyota.spp	native
Gelid.spp	Gelid.spp	native
Gelidiella.acerosa	Gelid.spp	native
Gelidiopsis.intricata	Gelid.spp	native
Gelidiopsis.spp	Gelid.spp	native
Gelidium.pusillum	Gelid.spp	native
Gracilaria.native	Gracilaria.native	native
Gracilaria.salicornia	Gracilaria.salicornia	non-native.invasive
Grateloupia.spp	Grateloupia.spp	native
Griffithsia.spp	Griffithsia.spp	native
Halimeda.discoidea	Halimeda.discoidea	native
Hydroclathrus	Hydroclathrus.clathratus	native
Hydroclathrus.clathratus	Hydroclathrus.clathratus	native
Hypnea.musciformis	Hypnea.musciformis	non-native.invasive
Hypnea.cervicornis	Hypnea.spp	native
Hypnea.chordacea	Hypnea.spp	native
Hypnea.spinella	Hypnea.spp	native
Hypnea.spp	Hypnea.spp	native
Chondrophycus.parvipapillatu	Laurencia.spp	native
Laurencia.dendroidea	Laurencia.spp	native
Laurencia.dotyi	Laurencia.spp	native
Laurencia.mcdermidiae	Laurencia.spp	native
Laurencia.spp	Laurencia.spp	native
Leptolyngbya.crosbyana	Leptolyngbya.crosbyana	native
Lobophora.spp	Lobophora.variegata	native
Lobophora.variegata	Lobophora.variegata	native
Martensia.spp	Martensia.spp	native
Microdictyon.setchellianum	Microdictyon.setchellianum	native
Neomeris.spp	Neomeris.spp	native
Padina.spp	Padina.spp	native
Palisada.parvipapillata	Palisada.parvipapillata	native
Parvocaulis.parvulus	Parvocaulis.parvulus	native
Pterocladiella.caeruluscens	Pterocladiella.spp	native
Pterocladiella.capillacea	Pterocladiella.spp	native
Pterocladiella.spp	Pterocladiella.spp	native
Akalaphycus.setchelliae	Red.Calcifying.Branching.Algae	native
Articulated.Corallines	Red.Calcifying.Branching.Algae	native
Dichotomaria.marginata	Red.Calcifying.Branching.Algae	native
Galaxaura.spp	Red.Calcifying.Branching.Algae	native
Jania	Red.Calcifying.Branching.Algae	native
Liagora.spp	Red.Calcifying.Branching.Algae	native

C

Initial Identification	Reclassified by Taxa	Classification
Trichogloea.spp	Trichogloea.spp	native
Turbinaria.ornata	Turbinaria.ornata	native.dominant
Turf	Turf	native.dominant
Enteromorpha.spp	Ulva.spp	native.dominant
Ulva.fasciata	Ulva.spp	native.dominant
Ulva.flexuosa	Ulva.spp	native.dominant
Ulva.reticulata	Ulva.spp	native.dominant
Ulva.spp	Ulva.spp	native.dominant
other.algae	Unknown.Algae	unknown
Unknown.Brown	Unknown.Brown.Algae	unknown
Unknown.Green	Unknown.Green.Algae	unknown
Unknown.Fuzzy	Unknown.Red.Algae	unknown
Unknown.Red	Unknown.Red.Algae	unknown
Valonia.spp	Valonia.spp	native
Actinopyga.mauritana	Actinopyga.spp	native
Actinopyga.obesa	Actinopyga.spp	native
Actinopyga.varians	Actinopyga.spp	native
Amphipod	Amphipod.spp	native
Aiptasia.pulchella	Anemone.spp	native
Anthopleura.nigrescens	Anemone.spp	native
Aplysia.spp	Aplysia.spp	native
Barnacles	Barnacles	native
Nesochthamalus.intertextus	Barnacles	native
Bleached.coral	Bleached.coral	unknown
Brachidontes.crebristriatus	Brachidontes.crebristriatus	native
Brittle.Star	Brittle.Star	native
Ophiocoma.brevipes	Brittle.Star	native
Ophiocoma.erinaceus	Brittle.Star	native
Calcinus.elegans	Calcinus.spp	native
Calcinus.laevimanus	Calcinus.spp	native
Calcinus.latens	Calcinus.spp	native
Calcinus.seurati	Calcinus.spp	native
Cellana.exarata	Cellana.spp	native
Cellana.sandwicensis	Cellana.spp	native
Chthamalus.intertextus	Chthamalus.intertextus	non-native
Colobocentrotus.atratus	Colobocentrotus.atratus	native
Conus.ebraeus	Conus.spp	native
Conus.sp.	Conus.spp	native
Conus.spp	Conus.spp	native
Crab	Crab	unknown
Cypraea.caputserpentis	Cypraea.spp	native
Cypraea.mauritana	Cypraea.spp	native

D

Initial Identification	Reclassified by Taxa	Classification
Sargassum.polyphyllum	Sargassum.polyphyllum	native.dominant
Sargassum.aquifolium	Sargassum.spp	native
Sargassum.spp	Sargassum.spp	native
Sphacelaria.spp	Sphacelaria.spp	native
Symploca.hydnoides	Symploca.hydnoides	native
Fireworm	Fireworm	native
Haminoea.cymbalum	Haminoea.cymbalum	native
Hermit.crab	Hermit.crab	native
Holothuria.atra	Holothuria.spp	native
Holothuria.cinerascens	Holothuria.spp	native
Holothuria.difficilis	Holothuria.spp	native
Holothuria.hilla	Holothuria.spp	native
Holothuria.parda	Holothuria.spp	native
Holothuria.spp	Holothuria.spp	native
Isognomon.californicum	Isognomon.spp	native
Isognomon.perna	Isognomon.spp	native
Isognomon.spp	Isognomon.spp	native
Littoraria.pintado	Littorinidae.spp	native
Littorinidae	Littorinidae.spp	native
Loimia.medusa	Loimia.medusa	native
Mauritia.mauritana	Mauritia.mauritana	native
Monetaria.caputserpentis	Cypraea.spp	native
Montipora.capitata	Montipora.spp	native
Montipora.flabellata	Montipora.spp	native
Montipora.spp	Montipora.spp	native
Morula.granulata	Morula.spp	native
Morula.spp	Morula.spp	native
Morula.uva	Morula.spp	native
Nerita.picea	Nerita.picea	native
Nodilittorina.hawaiiensis	Nodilittorina.hawaiiensis	native
Onchidium.verruculatum	Onchidium.verruculatum	native
Peasiella.tantilla	Peasiella.tantilla	native
Pennaria.disticha	Pennaria.disticha	non-native
Pocillopora.damicornis	Pocillopora.spp	native
Pocillopora.meandrina	Pocillopora.spp	native
Pocillopora.spp	Pocillopora.spp	native
Porites.lobata	Porites.spp	native
Porites.spp	Porites.spp	native
Siphonaria.normalis	Siphonaria.normalis	native
Spirobranchus.gigateus	Spirobranhus.gigateus	native
Sponge	Sponge	unknown
Stomadopod	Stomadopod	unknown

E

Initial Identification	Reclassified by Taxa	Classification
Cypraea.spp	Cypraea.spp	native
Dendropoma.gregaria	Dendropoma.gregaria	native
Diadema.paucispinum	Diadema.paucispinum	native
Drupa.morum	Drupa.morum	native
Echinolittorina.hawaiiensis	Echinolittorina.hawaiiensis	native
Echinometra.mathaei	Echinometra.spp	native
Echinometra.oblonga	Echinometra.spp	native
Echinometra.spp	Echinometra.spp	native
Echinothrix.calamaris	Echinothrix.spp	native
Echinothrix.spp	Echinothrix.spp	native
Portieria.hornemannii	Red.Calcifying.Branching.Alga	native
Dasya.spp	Red.Fuzzy.Algae	native
Spyridia.filamentosa	Red.Fuzzy.Algae	native
Wrangelia.elegantissima	Red.Fuzzy.Algae	native
Rhodymenia.sp	Rhodymenia.sp	native
Stylocheilus.striatus	Stylocheilus.striatus	native
Tripneustes.gratilla	Tripneustes.gratilla	native
Coral.spp	Unknown.Coral	unknown
unidentified.coral.sp	Unknown.Coral	unknown
other.inverts	Unknown.Inverebrate	unknown
Unknown.Polychaet	Unknown.Polychaet	unknown
other.whelks	Unknown.Whelk	unknown
Snails	Unknown.Whelk	unknown
Unknown.snails	Unknown.Whelk	unknown
Serpulorbis	Vermetidae.spp	native
Thylacodes.variabilis	Vermetidae.spp	native
Vermetidae	Vermetidae.spp	native
Palythoa.caesia	Zooanthid.spp	native
Protopalythoa.spp	Zooanthid.spp	native
Zoanthid.spp	Zooanthid.spp	native
