# Supplementary material for: Spatial variation and antecedent sea surface temperature conditions influence Hawaiian intertidal community structure
Source: PLoS One. 2023 Jun 2;18(6):e0286136. doi: 10.1371/journal.pone.0286136 (PMC10237483; doi:10.1371/journal.pone.0286136)
Supplement: S3 Table — (PDF) [file pone.0286136.s005.pdf]

Diamond Head

	value	NRT fit	NRT lower	NRT upper	CI
REP min	296.28	296.299	295.9693	296.6286	0.3297
REP max	302	301.9485	301.6187	302.2784	0.3298
REP mean	298.7838	298.7719	298.4425	299.1014	0.3294

	value	REP fit	REP lower	REP upper	CI
NRT min	296.3	296.3408	296.0111	296.6706	0.3297
NRT max	302.2	302.1717	301.8417	302.5016	0.3300
NRT mean	298.7719	297.7955	297.4659	298.1251	0.3296

Sand Island

	value	NRT fit	NRT lower	NRT upper	CI
REP min	296	296.0211	295.7619	296.2802	0.2592
REP max	302.24	302.1563	301.8969	302.4156	0.2594
REP mean	298.6221	298.5991	298.3401	298.8581	0.259

	value	REP fit	REP lower	REP upper	CI
NRT min	296.04	296.0566	295.7949	296.3183	0.2617
NRT max	302.19	302.2219	301.96	302.4837	0.2619
NRT mean	298.5991	298.6221	298.3605	298.8836	0.2616

Barber's Point

	value	NRT fit	NRT lower	NRT upper	CI
REP min	296.27	296.2876	295.9352	296.64	0.3524
REP max	302.24	302.0528	301.7003	302.4053	0.3525
REP mean	299.0378	298.9604	298.6083	299.3126	0.3521

	value	REP fit	REP lower	REP upper	CI
NRT min	296.54	296.5982	296.2383	296.9582	0.3599
NRT max	302.21	302.313	301.9529	302.6732	0.3601
NRT mean	298.9604	299.0378	298.678	299.3975	0.3598

**Sandy Beach**					
	value	NRT fit	NRT lower	NRT upper	CI
REP min	296.11	296.1273	295.8381	296.4165	0.2892
REP max	302.22	302.1308	301.8414	302.4202	0.2894
REP mean	298.6732	298.6458	298.3568	298.9349	0.289
	value	REP fit	REP lower	REP upper	CI
NRT min	296.12	296.1486	295.8569	296.4403	0.2917
NRT max	302.25	302.2756	301.9837	302.5675	0.2919
NRT mean	298.6459	298.6733	298.3817	298.9648	0.2916

**‘Ewa Beach**					
	value	NRT fit	NRT lower	NRT upper	CI
REP min	296.25	296.268	295.9062	296.6298	0.3618
REP max	302.21	302.0425	301.6806	302.4044	0.3619
REP mean	299.0271	298.9586	298.5971	299.3202	0.3615
	value	REP fit	REP lower	REP upper	CI
NRT min	296.55	296.6104	296.2422	296.9785	0.3682
NRT max	302.21	302.2894	301.9211	302.6577	0.3683
NRT mean	298.9587	299.0272	298.6592	299.3951	0.368

**Mā‘ili Point**					
	value	NRT fit	NRT lower	NRT upper	CI
REP min	296.57	296.5131	296.1507	296.8756	0.3624
REP max	301.89	301.7234	301.3608	302.0859	0.3626
REP mean	299.0447	298.9368	298.5745	299.2991	0.3623
	value	REP fit	REP lower	REP upper	CI
NRT min	296.55	296.6755	296.3106	297.0404	0.3649
NRT max	302.19	302.274	301.9089	302.6391	0.3651
NRT mean	298.9368	299.0447	298.68	299.4095	0.3647

**Waipu‘ilani**					
	value	NRT fit	NRT lower	NRT upper	CI
REP min	296.54	296.4922	296.0464	296.9381	0.4458
REP max	301.78	301.7811	301.3351	302.2271	0.446
REP mean	298.8156	298.7891	298.3435	299.2346	0.4456
	value	REP fit	REP lower	REP upper	CI
NRT min	296.04	296.2147	295.7829	296.6464	0.4318
NRT max	302.66	302.478	302.0459	302.91	0.4321
NRT mean	298.7891	298.8156	298.3842	299.247	0.4314

**Wai‘opae**					
	value	NRT fit	NRT lower	NRT upper	CI
REP min	296.16	296.0573	295.6131	296.5015	0.4442
REP max	301.35	301.3755	300.9311	301.8198	0.4444
REP mean	298.5797	298.5368	298.0928	298.9807	0.444
	value	REP fit	REP lower	REP upper	CI
NRT min	295.45	295.7187	295.296	296.1413	0.4227
NRT max	301.99	301.7804	301.3577	302.2032	0.4227
NRT mean	298.5367	298.5796	298.1575	299.0018	0.4221

**Hilo Yacht Club**					
	value	NRT fit	NRT lower	NRT upper	CI
REP min	296.12	296.0288	295.5677	296.49	0.4611
REP max	301.11	301.0419	300.5808	301.5031	0.4611
REP mean	298.5865	298.5068	298.0459	298.9676	0.4609
	value	REP fit	REP lower	REP upper	CI
NRT min	295.43	295.6817	295.2345	296.1289	0.4472
NRT max	301.96	301.8468	301.3995	302.2941	0.4473
NRT mean	298.5068	298.5865	298.1398	299.0333	0.4467

Onekahakaha

	value	NRT fit	NRT lower	NRT upper	CI
NRT min	296.12	296.0288	295.5677	296.49	0.4611
NRT max	301.11	301.0419	300.5808	301.5031	0.4611
NRT mean	298.5865	298.5068	298.0459	298.9676	0.4609
	value	REP fit	REP lower	REP upper	CI
REP min	295.43	295.6817	295.2345	296.1289	0.4472
REP max	301.96	301.8468	301.3995	302.2941	0.4473
REP mean	298.5068	298.5865	298.1398	299.0333	0.4467

Morris Point

	value	NRT fit	NRT lower	NRT upper	CI
NRT min	295.8	295.758	295.3493	296.1667	0.4087
NRT max	301.44	301.4258	301.017	301.8346	0.4088
NRT mean	298.5028	298.4741	298.0657	298.8825	0.4084
	value	REP fit	REP lower	REP upper	CI
REP min	295.85	295.9921	295.5933	296.3908	0.3988
REP max	302.06	301.9338	301.5347	302.3328	0.3991
REP mean	298.4741	298.5028	298.1043	298.9013	0.3985

Mapulehu

	value	NRT fit	NRT lower	NRT upper	CI
NRT min	295.81	295.8498	295.4304	296.2692	0.4194
NRT max	301.49	301.5693	301.1499	301.9888	0.4194
NRT mean	298.561	298.6199	298.2009	299.039	0.419
	value	REP fit	REP lower	REP upper	CI
REP min	295.96	296.0261	295.6181	296.4341	0.408
REP max	302.22	301.9918	301.5836	302.4001	0.4082
REP mean	298.62	298.5611	298.1533	298.9688	0.4078
