# Supplementary material for: Spatial variation and antecedent sea surface temperature conditions influence Hawaiian intertidal community structure
Source: PLoS One. 2023 Jun 2;18(6):e0286136. doi: 10.1371/journal.pone.0286136 (PMC10237483; doi:10.1371/journal.pone.0286136)
Supplement: S5 Table — (A) Summary statistics for mean yearly SST across the MHI from 2004–2019. Dates in gray are from the first sampling period (2004–2011) and dates in white are from the second sampling period (2012–2019). (B) Results from two-tailed t-test comparing monthly sea surface temperature (SST) statistics from 2004–2011 to 2012–2019 for the Main Hawaiian Islands. (PDF) [file pone.0286136.s007.pdf]

**A**

| # | Year | Mean | Min | Max | SD | Variance |
|---|---|---|---|---|---|---|
| 1 | 2004 | 26.17735 | 24.67 | 27.87 | 0.973832 | 0.948348 |
| 2 | 2005 | 25.84721 | 24.2 | 27.53 | 0.8998 | 0.80964 |
| 3 | 2006 | 25.58123 | 24.06 | 27.35 | 1.018965 | 1.03829 |
| 4 | 2007 | 25.60 | 24.08 | 27.10 | 0.890 | 0.792 |
| 5 | 2008 | 25.46 | 23.91 | 26.91 | 0.885 | 0.782 |
| 6 | 2009 | 25.46 | 23.02 | 27.25 | 1.281 | 1.641 |
| 7 | 2010 | 25.31 | 23.93 | 26.71 | 0.763 | 0.582 |
| 8 | 2011 | 25.46 | 24.19 | 26.66 | 0.634 | 0.403 |
| 9 | 2012 | 25.01 | 23.88 | 26.45 | 0.779 | 0.607 |
| 10 | 2013 | 25.60 | 23.52 | 27.22 | 1.144 | 1.308 |
| 11 | 2014 | 26.07 | 24.60 | 28.32 | 1.090 | 1.187 |
| 12 | 2015 | 26.23 | 24.20 | 28.91 | 1.316 | 1.732 |
| 13 | 2016 | 26.08 | 24.59 | 27.79 | 0.910 | 0.827 |
| 14 | 2017 | 26.11 | 24.42 | 27.97 | 0.997 | 0.994 |
| 15 | 2018 | 25.89 | 23.90 | 27.76 | 1.125 | 1.266 |
| 16 | 2019 | 26.27 | 23.57 | 28.16 | 1.482 | 2.197 |

**B**

| Summary Statistic | 2004-2011 Average | 2012-2019 Average | p-value | t-stat | DF |
|---|---|---|---|---|---|
| Mean | 25.6 | 25.9 | 0.06 | -1.91 | 190 |
| Minimum | 25.3 | 25.6 | 0.13 | -1.50 | 190 |
| Maximum | 25.9 | 26.3 | 0.04 | -2.06 | 190 |
| Variance | 0.04 | 0.05 | 0.32 | -1.00 | 190 |
