# Supplementary material for: Spatial variation and antecedent sea surface temperature conditions influence Hawaiian intertidal community structure
Source: PLoS One. 2023 Jun 2;18(6):e0286136. doi: 10.1371/journal.pone.0286136 (PMC10237483; doi:10.1371/journal.pone.0286136)
Supplement: S6 Table — Additionally, the relationship between SST and point count abundance was also assessed for the 10 most abundant taxa among all 12 sites cumulatively. All analyses were conducted in climwin via negative binomial regression. Grouped by taxa, only statistically significant relationships are shown. (PDF) [file pone.0286136.s008.pdf]

Island	Location	Taxa	Mean Point Count	Std. Dev	Climate Statistic	Δ in point count per +0.1°C	95% CI	p-value	Window Open	Window Close
MHI	All Twelve Intertidal Sites	Acanthophora.spicifera	0.71	1.53	min	(+) 18.7%	6.62%-32.1%	0.002	12	0
Oahu	Maili Point, Oʻahu	Acanthophora spicifera	4.45	5.00	min	(+) 35.8%	27.7%-45.1%	<0.001	11	2
Oahu	Barber's Point, Oʻahu	Acanthophora spicifera	1.39	3.54	mean	(+) 29.9%	16.7%-45.5%	<0.001	8	7
Oahu	Ewa Beach, Oʻahu	Acanthophora spicifera	1.24	3.20	min	(+) 26.6%	20.3%-33.3%	<0.001	12	2
Molokai	Mapulehu, Molokaʻi	Acanthophora spicifera	1.24	2.78	min	(+) 43.9%	25.5%-66.1%	<0.001	11	4
Maui	Waipuʻilani, Maui	Acanthophora spicifera	1.64	3.38	max	(+) 28.5%	18.7%-39.4%	<0.001	3	1
MHI	All Twelve Intertidal Sites	Brown.Crust	0.30	1.29	max	(+) 14.6%	14.5%-14.8%	<0.001	9	5
Oahu	Sand Island, Oʻahu	Brown Crust	0.47	1.94	min	(+) 42.6%	31.6%-55.9%	0.002	9	0
Maui	Waipuʻilani, Maui	Brown Crust	0.30	1.51	min	(-) 62.3%	35.7%-82.6%	<0.001	10	5
Hawaii	Waiʻopae, Hawaiʻi	Brown Crust	3.40	7.20	mean	(+) 60.6%	40.1%-88.2%	<0.001	11	3
Hawaii	Onekahakaha, Hawaiʻi	Brown Crust	0.32	1.43	min	(+) 305%	207%-484%	<0.001	11	0
Molokai	Mapulehu, Molokaʻi	Cladophora.spp	1.29	2.23	min	(+) 30.7%	22.0%-40.9%	<0.001	10	4
MHI	All Twelve Intertidal Sites	Crustose.coralline.algae	1.00	1.69	max	(+) 4.87%	0.17%-9.78%	0.042	7	1
Oahu	Sandy Beach, Oʻahu	Crustose coralline algae	0.61	2.07	min	(+) 31.5%	12.6%-56.7%	<0.001	4	0
Oahu	Sand Island, Oʻahu	Crustose coralline algae	0.50	1.42	max	(+) 18.9%	13.5%-24.9%	<0.001	2	0
Molokai	Mapulehu, Molokaʻi	Crustose coralline algae	2.10	4.85	min	(-) 29.7%	21.6%-38.5%	<0.001	11	3
Maui	Waipuʻilani, Maui	Crustose coralline algae	3.83	5.27	min	(+) 42.7%	31.9%-54.4%	<0.001	12	1
Hawaii	Waiʻopae, Hawaiʻi	Crustose coralline algae	2.35	3.19	min	(+) 460%	261%-840%	<0.001	10	6
Hawaii	Hilo Yacht Club, Hawaiʻi	Crustose coralline algae	2.04	4.43	mean	(-) 30.9%	16.9%-42.4%	<0.001	3	1
Hawaii	Onekahakaha, Hawaiʻi	Crustose coralline algae	1.89	4.64	NA	(+) 12.5%	3.82%-21.7%	0.003	1	1
MHI	All Twelve Intertidal Sites	Dendropoma.gregaria	0.24	0.66	min	(-) 13.27%	2.01%-23.2%	0.023	12	3
Oahu	Barber's Point, Oʻahu	Dendropoma gregaria	0.51	2.14	min	(+) 21.7%	9.12%-36.1%	<0.001	10	5
Oahu	Ewa Beach, Oʻahu	Dictyota spp	0.29	1.48	min	(+) 15.2%	8.78%-22.2%	<0.001	12	5
MHI	All Twelve Intertidal Sites	Gracilaria.salicornia	0.19	0.50	min	(+) 14.78	14.7%-14.9%	<0.001	12	0
Hawaii	Onekahakaha, Hawaiʻi	Gracilaria salicornia	1.21	2.75	min	(+) 35.1%	22.1%-50.3%	<0.001	3	0

Island	Location	Taxa	Mean Point Count	Std. Dev	Climate Statistic	Δ in point count per +0.1°C	95% CI	p-value	Window Open	Window Close
Hawaii	Hilo Yacht Club, Hawai'i	Nerita.spp	0.47	1.90	min	(+) 328%	176%-875%	0.005	9	7
MHI	All Twelve Intertidal Sites	Padina.spp	3.14	3.83	min	(+) 4.32%	0.47-9.36%	0.078	11	5
Oahu	Ewa Beach, O'ahu	Padina spp	6.92	6.90	max	(+) 4.87%	2.93%-6.85%	<0.001	1	0
Oahu	Barber's Point, O'ahu	Padina spp	6.20	6.07	mean	(-) 4.84%	2.55%-7.09%	<0.001	10	9
Oahu	Diamond Head, O'ahu	Padina spp	5.23	6.64	min	(+) 6.75%	3.96%-9.61%	<0.001	12	4
Oahu	Sand Island, O'ahu	Padina spp	1.02	2.50	max	(-) 18.6%%	14.8%-22.3%	<0.001	1	0
Oahu	Sandy Beach, O'ahu	Padina spp	0.35	1.28	min	(+) 30.3%	13.9%-55.3%	0.001	11	5
Molokai	Mapulehu, Moloka'i	Padina spp	1.31	3.39	min	(-) 35.6%	29.1%-42.4%	<0.001	11	5
Oahu	Maili Point, O'ahu	Pterocladiella.spp	0.77	2.55	max	(+) 12.5%	5.25%-20.9%	<0.001	2	1
Oahu	Sandy Beach, O'ahu	Red Calcifying Branching Algae	0.31	1.37	max	(+) 40.8%	23.5%-62.5%	<0.001	9	8
Oahu	Sand Island, O'ahu	Red Calcifying Branching Algae	0.20	1.58	min	(-) 25.0%	17.1%-33.8%	<0.001	9	6
MHI	All Twelve Intertidal Sites	Sargassum.spp	0.25	0.66	min	(-) 5.738%	0.59%-10.6%	0.029	12	2
Oahu	Diamond Head, O'ahu	Sargassum spp	0.84	2.95	mean	(-) 10.5%	4.79%-16.0%	0.002	12	3
MHI	All Twelve Intertidal Sites	Turbinaria.ornata	0.25	0.63	min	(-) 14.17%	3.22%-23.9%	0.013	12	2
Oahu	Diamond Head, O'ahu	Turbinaria ornata	0.93	3.17	min	(-) 18.5%	13.2%-23.5%	<0.001	11	0
MHI	All Twelve Intertidal Sites	Turf	5.55	4.99	min	(-) 3.47%	0.547-6.31%	0.020	10	4
Oahu	Sand Island, O'ahu	Turf Algae	8.83	8.53	NA	(+) 10.6%	7.25%-14.2%	<0.001	0	0
Oahu	Maili Point, O'ahu	Turf Algae	4.40	5.80	mean	(+) 36.4%	23.3%-51.0%	<0.001	9	4
Oahu	Diamond Head, O'ahu	Turf Algae	3.38	5.70	min	(-) 7.08%	4.55%-9.67%	<0.001	8	5
Oahu	Ewa Beach, O'ahu	Turf Algae	2.97	4.93	max	(+) 5.1%	2.97%-7.31%	<0.001	4	0
Oahu	Barber's Point, O'ahu	Turf Algae	2.19	4.61	mean	(-) 26.3%	15.7%-36.2%	<0.001	12	0
Maui	Waipu'ilani, Maui	Turf Algae	1.56	3.01	NA	(+) 8.18%	4.74%-11.9%	<0.001	6	6
Hawaii	Wai'opae, Hawai'i	Turf Algae	12.28	8.04	NA	(-) 7.86%	10.1%-5.53%	<0.001	6	6
Hawaii	Onekahakaha, Hawai'i	Turf Algae	11.01	8.58	mean	(-) 16.2%	11.0%-21.0%	<0.001	9	4
Hawaii	Hilo Yacht Club, Hawai'i	Turf Algae	7.27	8.06	min	(+) 39.5%	18.5%-64.1%	<0.001	9	6
Oahu	Diamond Head, O'ahu	Unknown Red Algae	0.57	2.48	min	(+) 55.6%	40.9%-72.8%	<0.001	11	0
Hawaii	Onekahakaha, Hawai'i	Vermetidae spp	0.32	1.61	max	(-) 13.8%	4.59%-23.0%	0.014	2	0
