# Supplementary material for: Spatial variation and antecedent sea surface temperature conditions influence Hawaiian intertidal community structure
Source: PLoS One. 2023 Jun 2;18(6):e0286136. doi: 10.1371/journal.pone.0286136 (PMC10237483; doi:10.1371/journal.pone.0286136)
Supplement: S7 Table — Out of 12 intertidal sites assessed, only the eight found to have a statistically significant relationship between diversity and SST warming are shown. (PDF) [file pone.0286136.s009.pdf]

Location	# Transects	Avg. Diversity Index Value	Std. Dev	Climate Statistic	Δ in diversity per 1°C	95% CI	p-value	Window Open	Window Close
Barber's Point, O‘ahu	68	0.579	± 0.222	NA	(-)0.165	0.043-0.286	0.008	7	7
Diamond Head, O‘ahu	133	0.666	± 0.168	max	(-) 0.117	0.066-0.167	<0.001	12	10
Sand Island, O‘ahu	106	0.342	± 0.252	min	(-) 0.120	0.0457-0.194	0.002	12	11
Sandy Beach, O‘ahu	31	0.409	± 0.268	min	(+) 0.147	0.018-0.276	0.03	2	0
Ewa Beach, O‘ahu	121	0.540	± 0.207	min	(+) 0.081	0.011-0.152	0.02	9	3
Waipu‘ilani, Maui	40	0.517	± 0.232	max	(-) 0.230	0.073-0.388	0.005	8	7
Wai‘opae, Hawai‘i	19	0.388	± 0.226	max	(-) 0.577	0.253-0.900	0.002	3	2
Onekahakaha, Hawai‘i	46	0.422	± 0.223	mean	(+) 0.622	0.334-0.909	<0.001	9	4
