# Supplementary material for: Spatial variation and antecedent sea surface temperature conditions influence Hawaiian intertidal community structure
Source: PLoS One. 2023 Jun 2;18(6):e0286136. doi: 10.1371/journal.pone.0286136 (PMC10237483; doi:10.1371/journal.pone.0286136)
Supplement: Attachment — Submitted filename: ResponsetoReviewers_PONE-D-22-26540_Ward.docx [file pone.0286136.s010.docx]

Dear Dr. Ummerhofer,

Thank you for serving as the handling editor and for reviewing our manuscript. Please see below for a point-by-point response to all comments. In short, we made all changes and clarifications requested. Changes are highlighted below, in red.

**Journal Requirements**

1. Please ensure that your manuscript meets PLOS ONE's style requirements, including those for file naming. The PLOS ONE style templates can be found at

https://journals.plos.org/plosone/s/file?id=wjVg/PLOSOne_formatting_sample_main_body.pdf and

<https://journals.plos.org/plosone/s/file?id=ba62/PLOSOne_formatting_sample_title_authors_affiliations.pdf>"

>>We completed a review of all style requirements. File names were edited to include the file type at the end of the name (i.e., previously: “Fig1”, corrected: “Fig1.tiff”)

2. In your Methods section, please provide additional information regarding the permits you obtained for the work. Please ensure you have included the full name of the authority that approved the field site access and, if no permits were required, a brief statement explaining why.

>>Completed. A brief statement was added to the end of the Methods section *OPIHI Sampling Methods* which states: “Permits were not required for the recording of these biological data, as no organisms were collected or harmed, and all beaches and shorelines in Hawaiʻi are in the public domain.”

3. "We note that Figure (1) in your submission contain map images which may be copyrighted. All PLOS content is published under the Creative Commons Attribution License (CC BY 4.0), which means that the manuscript, images, and Supporting Information files will be freely available online, and any third party is permitted to access, download, copy, distribute, and use these materials in any way, even commercially, with proper attribution. For these reasons, we cannot publish previously copyrighted maps or satellite images created using proprietary data, such as Google software (Google Maps, Street View, and Earth). For more information, see our copyright guidelines: http://journals.plos.org/plosone/s/licenses-and-copyright.

We require you to either (1) present written permission from the copyright holder to publish these figures specifically under the CC BY 4.0 license, or (2) remove the figures from your submission.

>> We have received written permission from the copyright holder of Figure 1 (Dean Lodes, Director of Marketing and Publication Services with the Curriculum Research & Development Group at the University of Hawai‘i – Mānoa) to publish the image under the CC BY 4.0 license. The completed content permission form will be uploaded as an “Other” file with the resubmission.

**Reviewer #1**

This was a very nicely written, pertinent and interesting paper and i enjoyed reading it. There are just a few formatting errors though that need correction as follows (indicated by line number):

>> Thank you very kindly for your positive comments and helpful edits All suggested changes have been made to the manuscript and are reflected in the updated draft and document with tracked edits.

1. 30 - km2, should be a superscript

>> Corrected

2. All though the paper you are inconsistent in the rule as to use of numerals or words for numbers. The usual rule is the use words for numbers up to nine and then numerals for 10 and upwards. You do generally follow this, but repeatedly talk about twelve (should be 12) sites (lines 30 151, 193 etc many times) and sixteen (should be 16) years (e.g. lines 206 266 and many other places). You also have ten not 10 on lines 296, 298, but correctly 12 on line 513 for example! In the reverse way you have several number under 10 on lines 344 and these should be given as words! This must at least be consistent though entire paper whatever rule you follow.

>> Corrected throughout the manuscript

3. 422, also 446, 452,575 surely this should read '21st century'

>> Corrected

4. 650 species name must have a lower case

>> Corrected

5. 753, 787 Do not capitalise the words in the titles of journal articles ( even if this was done in the original journal, as some do do)

>> Corrected

6. 766 there are 2 Smith refs here, the second one needs a new line and new number

>> Corrected- the second Smith reference was inserted accidentally.

7. 776, 788 you need to abbreviate this journal title for consistency

>> Corrected

**Reviewer #2:**

>>Thank you for your thoughtful and thorough comments on this manuscript. We have addressed each of your requested changes below:

1. This paper reports a study examining changes in Hawaiian shoreline communities in relation to sea surface temperature. The paper potentially offers new and useful information on the ecology of these systems. However, in its current form, the paper is not reviewable. Although the core of the paper is the abundance of shoreline species, key figures with these data are missing in my copy, specifically Supplementary Tables 6 and 7 and Figure 6.

>> Our sincerest apologies for the omission of these tables and figure. It was an error that occurred during the final formatting and submission, and the updated manuscript is uploaded to include the missing items. They are also included below for your convenience, along with their figure/table legends.

**Figure 6. Generalized Dissimilarity Model-Fitted Partial Regressions.** Generalized dissimilarity model-fitted partial regressions for all variables found to significantly explain community dissimilarity. Each curve’s maximum height represents the total amount of community dissimilarity associated with the variable and the slope of the lines represents the rate at which community dissimilarity increases along the variable’s gradient. Model results explained 45.1% of the deviance in community composition between sites (p<0.05). **(A)** Average SST from the first half of this study’s timeframe (2004-2011) was the most influential variable for explaining dissimilarity between intertidal communities (magnitude = 65.4, p<0.05; red line), followed by **(B)** mean SST for the entire study period (2004-2019; magnitude = 24.6, p=0.04; orange line), and then by **(C)** geographic distance (magnitude = 1.94, p<0.05; green line).

**S6 Table.** The relationship between SST and point-count abundance for each of the five most abundant taxa within 12 intertidal sites across the Main Hawaiian Islands (MHI). Additionally, the relationship between SST and point count abundance was also assessed for the 10 most abundant taxa among all 12 sites cumulatively. All analyses were conducted in *climwin* via negative binomial regression. Grouped by taxa, only statistically significant relationships are shown.

**S7 Table.** The relationship between SST and Simpson’s diversity (1-D) among 12 intertidal sites within the Main Hawaiian Islands, assessed via linear regression in *climwin*. Out of 12 intertidal sites assessed, only the eight found to have a statistically significant relationship between diversity and SST warming are shown.

2. Beyond missing figures, most figures in the paper only provide statistical summary lines/curves rather than the biological data themselves. It is essential that the underlying data also be provided so that the reader can assess the fits to the data. This is especially true of Figure 4, which offers relationships with the species abundance data, but is also true for Figs. 3 and 5.

>> We have updated figures 3-5 to include the biological and SST data behind the statistical summary lines/curves. Additionally, Figure 3 was updated to display the non-parametric LOESS curve fit, rather than the linear, to mirror the updated Figure 2. For your convenience, these figures are also provided below:

**Figure 3.** **Linear SST Relationships Trends across for the MHI and 12 Intertidal Study Sites, 2004—2019.** LOESS non-parametric curve fit of Linear SST relationships trends for the MHI collectively (black line; MHI) and the twelve12 intertidal study sites across the islands of Oʻahu, Hawaiʻi, Maui, and Molokaʻi from 2004-2019. All were found to have statistically significant (p<0.01) warming trends (R2 values by site: Diamond Head= 0.020, Sandy Beach = 0.022, Sand Island = 0.021, Barber's Point, = 0.027, Ewa Beach, = 0.027, Mā‘ili Point = 0.022, Wai‘ōpae = 0.034, Hilo Yacht Club, = 0.030, Onekahakaha = 0.030, Waipu‘ilani = 0.026, Mapulehu = 0.022, Morris Point = 0.020).

**Figure 4.** Negative binomial relationships between sea surface temperature (SST) and daily mean point-count abundance for the six most abundant taxa with statistically significant (p<0.05) relationships across the 12 intertidal sites within the Main Hawaiian Islands from 2004–2019. The negative binomial relationship modeled for each taxon (Table 2A) is indicated by the black line, 95% confidence intervals are in dark gray.

**Figure 5. Linear Relationship between Simpson’s Diversity and SST.** The linear relationship between Simpson’s Diversity and mean sea surface temperature (SST) 1-10 months prior to the sampling date for all twelve12 intertidal sites collectively across the islands of Oʻahu, Hawaiʻi, Maui, and Molokaʻi from 2004-2019. The linear regression is indicated by the black line, 95% confidence interval in dark gray.

3. More information in Figure 2 is also needed. Specifically, a linear temporal trend is provided, but visually the temporal patterns appears to exhibit an oscillatory pattern across the duration of the data (beyond the annual cycles). Perhaps there is a signal of a relationship to some large-scale oceanographic pattern such as the PDO that can be extracted, or perhaps using a non-linear regression with an annual cosine function, an unspecified cosine function on the order of 20 yr (make the period a variable to be fitted) and a linear function would clarify the magnitude of a potential trend. The concern is that a longer-period oscillation can look like a long-term trend and the pattern in the data indicates a distinctly non-linear trajectory through time. At the least, a non-parametric curve fit (LOESS, DWLS or similar) would be beneficial.

>> Thank you for this important feedback regarding our analyses and visualizations of SST trends. To investigate the non-linearity of SST over the study’s timeframe, we updated our SST analyses to include a LOESS non-parametric curve fit, as suggested. We then clarified the change in SST trends from 2004—2011 to 2012—2019. Additionally, we updated our manuscript to acknowledge the potential influence of larger oceanographic patterns, such as PDO, and argue the relatively limited timeframe of our study (16 years) makes it difficult to adequately address their possible impacts on SST trends in Hawaiʻi within and beyond the timeframe of this study. Below is the updated figure 2 and its corresponding legend.

**Figure 2. Monthly SST for the MHI, 2004–2019.** Mean monthly SST measurements (red line), LOESS non-parametric curve fit (dark blue line) and 95% confidence interval (light blue band), and linear regression line (dashed grey line) for the Main Hawaiian Islands, 2004–2019. SST data were produced by the Operational Sea Surface Temperature and Sea Ice Analysis product (OSTIA), obtained from the European Union’s Earth Observation Program, Copernicus (https://marine.copernicus.eu/).

**Reviewer #3**

This is a very timely and interesting study that does very well in incorporating citizen science. I found the methods to be rigorous and well explained, findings and interpretations to be justified, and the manuscript to be very well written. I normally have more comments and feedback to authors, but in this case there's not much more to say. I look forward to seeing this article published.

>>Thank you for your kind and generous comment. We are so glad you enjoyed our study.
