# Supplementary material for: Spatial variation and antecedent sea surface temperature conditions influence Hawaiian intertidal community structure
Source: PLoS One. 2023 Jun 2;18(6):e0286136. doi: 10.1371/journal.pone.0286136 (PMC10237483; doi:10.1371/journal.pone.0286136)
Supplement: Attachment — Submitted filename: ResponsetoReviewers_PONE-D-22-26540_Ward.docx [file pone.0286136.s011.docx]

Dear Dr. Ummerhofer,

Thank you for serving as the handling editor and for reviewing our manuscript. Please see below for a point-by-point response to reviewer comments. In short, we have addressed all reviewer comments and clarifications requested.

**Reviewer #2**

The authors have addressed some of my concerns regarding the figures and the inference of SST patterns. I would be happier if they had used a non-linear model to extract the annual and the possible longer term oscillation and then looked for a trend, but at least the non-parametric smoothing is available to get a sense of the pattern and this is noted in the text. I feel the inclusion of the data points on the graphs is especially valuable. From the perspective of the PLoS ONE review criteria, I feel the information provided and analyses are sufficient and the conclusions drawn are reasonable, but will raise a few more points for the authors to consider that might strengthen the paper further.

>> Thank you very kindly for your helpful and positive comments. Below we have addressed your new points of consideration. Additionally, all changes to the manuscript in the updated document are highlighted by utilizing “tracked changes.”

1. First, I do worry about the high likelihood of erroneously identifying a relationship that goes along with applying an approach that makes a massive number of different statistical tests of association to find the “best” temperature window to relate to species changes in time and space (statistical fishing expedition). Normally one would require a severe correction of alpha values to control study-wide error rates (as is done in genomics, for example)—or in the case of model selection used here, large differences in relative support. It seems to me virtually certain using this type of procedure that associations will be found, whether real or due to chance. This type of analysis is largely unavoidable for the authors to explore latent responses, and I think that this insight outweighs the downsides. More broadly this issue can be avoided if the analysis is considered exploratory, and the authors do a good job of not reading much into the patterns they report. But it wouldn’t hurt to discuss this issue a bit more and emphasize that the results are exploratory, ideally generating specific hypotheses/predictions to test more rigorously in the future.

>> Thank you for addressing this aspect of our research design. Indeed, in traditional frequentist statistical hypothesis testing a typical approach to the “multiple test problem” is to adjust the alpha-critical value (e.g., Bonferroni Correction) by the number of comparisons being made. In a model selection approach, as we employed here, the goodness-of-fit among alternative models to the data is calculated utilizing the Akaike Information Criterion derived from the Maximum Likelihood estimate, and penalizes models which use more independent variables (parameters) as a way of over-fitting (e.g., see Burnam and Anderson, 2002). In our case, this over-fitting based on number of models parameters is not an issue, as we are only testing for model fit between the observed temperature data and the biological response (either abundance or diversity). However, we agree with the reviewer that the *climwin* approach of testing for the fit of multiple temperature time-windows on either the abundance or diversity response variable creates many models for comparison. While we understand why this all-subset model selection approach can be viewed as a “fishing expedition”, the alternative of *a prior* identification of a sub-set of candidate models (temperature time-windows) is also problematic (e.g., Burnham and Anderson 2004; Dochtemann and Jenkins, 2010; Burnham et al., 2010), especially when prior work is not available to generate a meaningful subset of models for the relationship between SST and abundance/diversity of these Hawaiian intertidal taxa. Further, while our analyses do have an exploratory nature, we believe the insights gained from this approach provide a stepping stone for future hypothesis testing on the possible effects of SST in Hawaiian intertidal communities. In our discussion, we have now more clearly stated this point.

References cited

Burnham KP, Anderson DR (2002) Model selection and multimodel inference, 2nd edn.	Springer, New York

Burnham KP, Anderson DR (2004) Multimodel inference: understanding AIC and BIC in	model selection. Sociol Methods Res 33:261–304

Burnham KP, Anderson DR, Huyvaert KP (2010) AICc model selection in the ecological	and behavioral sciences: some background, observations and comparisons. Behav Ecol	Sociobiol. doi:10.1007/s00265-010-1029-6

Dochtermann NA, Jenkins SH (2010) Developing multiple hypotheses in behavioral	ecology. Behav Ecol Sociobiol. doi:10.1007/ s00265-010-1039-4

## Discussion

## Future Studies and Implications

…We acknowledge that, while our analyses assessing the relationship between SST and abundance or diversity have an exploratory nature, they provide a first foundation of insights into the influence of SST on Hawaiian intertidal communities, and we offer the following suggestions to further address these hypothesized relationships. Due to the detected significance of multi-year trends in antecedent SST on Hawaiian intertidal community structure, we predict continued lagging shifts in community composition will ~~likely~~ be observed as rates of warming continue to increase in both the near and distant future [3]…

2. Second, I continue to feel that there is missed opportunity in the paper to take advantage of the rich biological data set that makes this a noteworthy contribution. For example, why not carry out a PCA or other dimension reduction analysis on the Reclassified Taxa, tossing out the rarest taxa but retaining the others to get a broader sense of community change, and compare the dominant axes to SST? Other interesting relationships may be uncovered of broader functional groups (implied by the axis loading) that jointly change with temperature than can be revealed by just looking at the six most dominant taxa across sites. I was also curious about the response of native vs. invasive taxa, as there is some concern that the latter are disrupting Hawaiian shorelines (Hypnea musciformis, for example). One of course has to be careful here to qualify that invasives may be increasing simply through the dispersal/invasion process itself, but I do wonder if environmental change is a plausible contributor to driving invasion, although the dip in temperatures may help distinguish these effects.

>> Thank you for your thoughtful considerations on the use of biological data and potential analyses for expanding this research. We have several points of response to your comment and will try to address each of them thoroughly below:

a. “For example, why not carry out a PCA or other dimension reduction analysis on the Reclassified Taxa, tossing out the rarest taxa but retaining the others to get a broader sense of community change, and compare the dominant axes to SST? Other interesting relationships may be uncovered of broader functional groups (implied by the axis loading) that jointly change with temperature than can be revealed by just looking at the six most dominant taxa across sites.”

**Regarding community change and functional groups:** It may be helpful to point out that a comprehensive analysis of community change with these biological data was conducted in a recent publication by several of our co-authors (La Valle, Schaefer, et al. 2020; cited as #24 in our manuscript). In this publication, the question of how these biological communities have changed spatially (sites, islands, exposure type, substrate type) and temporally (years and decades) was addressed. They found changes in community composition and abundance across years were found to be site-specific and list the taxa that significantly changed in abundance (reported in Table 6 in La Valle et al.). Based on these prior findings, we opted not to conduct ordination analyses in the present study, as the dominant loading axes are likely to be both hyper-variable across all twelve focal study sites and driven by the most abundant taxa, which is already a primary focus of the present study. However, we agree that future work on additional aspects of community-level change is warranted and we make these suggestions more explicit in the Discusssion (please see below).

b. “I was also curious about the response of native vs. invasive taxa, as there is some concern that the latter are disrupting Hawaiian shorelines (Hypnea musciformis, for example). One of course has to be careful here to qualify that invasives may be increasing simply through the dispersal/invasion process itself, but I do wonder if environmental change is a plausible contributor to driving invasion, although the dip in temperatures may help distinguish these effects.”

**Regarding invasive taxa:** Thank you for highlighting this important point regarding invasive species and their possible impact on Hawaiian shorelines. Similarly, we were curious about the impacts of invasive taxa and conducted exploratory analyses to address this explicitly in prior drafts of the manuscript. However, a few key considerations led us to decide not focus this study on the invasive species in this data set. We found the presence and abundance of invasive species (i.e., *Hypnea musciformis*, *Gracilaria salicornia*) across all intertidal sites to be highly variable, with low to zero abundance at most sites, and since the OPIHI data are most appropriate for monitoring the more abundant species (Cox et al. 2012) we did not feel confident in including the analyses we conducted focused on the invasive species in the final version of the manuscript. However, we do note, that there were two instances where invasive algae were present as a dominant taxon within an intertidal site. At Onekahakaha, Hawai’i Island, *Gracilaria salicornia* was consistently detected as a highly abundant taxon. At this location, our site-level analysis found the abundance of *Gracilaria salicornia* to increase with increasing SSTs (S6 Table). In contrast, while *Hypnea musciformis* was found to be a highly abundant taxon at Waipuʻilani, Maui, we found no significant relationship between its abundance and SST, and we now point out these results in the revised manuscript (please see below).

**Results**

**Site-Level Responses**

        	Following biological data processing and filtering, a total of 116 algal and invertebrate taxa were identified across all 12 intertidal sites (S1 Table). The most abundant taxa often differed among the intertidal sites. The taxa which occurred the most frequently as one of the top five most abundant were turf algae (nine sites), crustose coralline algae (eight sites), *Padina* spp. (seven sites)*, Acanthophora spicifera* (six sites)*,* and brown crustose algae (five sites). Results of the *climwin* analyses revealed high variability in the relationship between SST and the daily mean point-count abundance for the five most abundant taxa across all sites and within each intertidal site (S6 Table). In particular, the strength and the direction of the relationship (reported as the % change in abundance per +0.1°C in SST) and the most strongly influencing SST variable (i.e., minimum, maximum, or mean monthly SST) were highly variable (S6 Table). There were two instances where invasive algae were present as a dominant taxon within an intertidal site. Notably, at the site Onekahakaha on Hawaiʻi Island, the invasive algae *Gracilaria salicornia* was found to be highly dominant and increased in abundance by 35.1% for every 0.1°C increase in minimum monthly SST (S6 Table). In contrast, while *Hypnea musciformis* was found to be a highly abundant taxon at Waipuʻilani, Maui, we found no significant relationship between its abundance and SST. Simpson’s diversity index was significantly related to SST at eight of the 12 sites, with no significant relationship at Māʻili Point, Hilo Yacht Club, Morris Point, and Mapulehu (S7 Table).

**Discussion**

**Site-Level Responses and Community Dissimilarity in Relationship to SST**

Previous studies have demonstrated that Hawaiian intertidal communities can vary greatly from one another, with interactions between local biotic and abiotic factors driving differences in community composition that are often difficult to predict [22,23,27]. This study’s site-level analyses support these prior general findings and further indicate high variability in community response to SST among intertidal sites (S6 Table; S7 Table). In addition, the response of Simpson’s diversity index to changing SST also differed among sites (S7 Table). While the abundance of the invasive algae, *Gracilaria salicornia* was found to have a positive relationship to SST at the site Onekahakaha on Hawaiʻi Island (S6 Table), additional investigation is required to understand how this relationship may be expressed across intertidal sites with differing local biotic and abiotic factors, as this invasive species may continue to extend its range. Although several of the same taxa were found to be abundant across multiple intertidal communities (e.g., turf algae, Padina spp., Acanthophora spicifera), their responses to warming often varied in both magnitude and direction between sites (S6 Table), underscoring the complexity of the drivers of change in these communities. …

**Future Studies and Implications**

…Future research incorporating these additional abiotic factors (among others) would be valuable. The significant influence of subtle differences in antecedent SST among intertidal sites on community dissimilarity (Fig 6) suggests that future intertidal studies should also be conducted at the finest temporal resolution possible. In addition, satellite-derived data, such as the OSTIA products used in this study [46], are an excellent resource for obtaining longer-term climatic data at a relatively fine spatial scale (~6 km^2^) when continual in-situ monitoring is not a possibility. To continue to better account for the role of SST and additional climatic variables on intertidal communities, it would be valuable to expand investigations to encompass longer timeframes (i.e., multiple years [33] to decades-long [10, 34, 35]) and to also assess the role of SST changes over a wider range of antecedent time-windows [51].

Continued funding and support for citizen and community-driven science initiatives, such as OPIHI [40], can provide a valuable, long-term source of high-quality intertidal ecological community data for sites across the state [19]. Future studies which build on our methods to explore the lagging influence of SST on intertidal community change, specific functional groups, and invasive taxa are important subsequent steps in this line of inquiry. In addition to temporal variation, as our results and the results of other recent studies have found, Hawaiian intertidal ecological communities can vary significantly across small spatial scales [23, 24] and in response to multiple biotic and abiotic ecological determinants through time. For this reason, whenever feasible, we recommend future analyses of the Hawaiian intertidal be conducted at multiple spatial and temporal scales to maximize confidence in detected trends.
